# Nafamostat mesylate has advantages as an anticoagulant for patients undergoing maintenance hemodialysis with conjunctival bleeding

**DOI:** 10.1093/ckj/sfae175

**Published:** 2024-06-13

**Authors:** Jian-Ying Tang, Lu Yang, Jing Wang, Ying Yang, Jun-Chen Li, Jiao Mu

**Affiliations:** Department of Nephrology, University-Town Hospital of Chongqing Medical University, Chongqing, China; Department of Nephrology, University-Town Hospital of Chongqing Medical University, Chongqing, China; Department of Nephrology, University-Town Hospital of Chongqing Medical University, Chongqing, China; Department of Nephrology, University-Town Hospital of Chongqing Medical University, Chongqing, China; Department of Nephrology, University-Town Hospital of Chongqing Medical University, Chongqing, China; Department of Nephrology, University-Town Hospital of Chongqing Medical University, Chongqing, China

To the Editor,

The process of hemodialysis (HD) activates the coagulation pathway and induces thrombosis [1]. The routine use of anticoagulants is necessary during HD to prevent coagulation. However, in patients with bleeding and those undergoing surgery, the use of conventional anticoagulants may increase the risk of bleeding and may be life-threatening.

Conjunctival bleeding has been observed in patients undergoing maintenance HD. Heparin-free dialysis or citrate anticoagulant dialysis were commonly used in the past, although the associations with coagulation blockages, thrombosis, and insufficient dialysis limit the use of these types of dialysis [2]. The use of anticoagulants may lead to increased conjunctival bleeding, prolonged recovery time, and increased psychological burden. The proper anticoagulant therapy must be administered.

Nafamostat mesylate (NM), a new anticoagulant, is characterized by its ability to be used in patients with bleeding or bleeding tendencies and can extend the lifespan of HD filters [3]. Most metabolites of NM are found in the bloodstream, with a small portion being metabolized in the liver [4]. NM has strong inhibitory activity against plasmin, thrombin, and active forms of coagulation factors (XIIa, Xa), without relying on antithrombin III; NM directly inhibits platelet aggregation and binds to fibrinogen by inhibiting the expression of activated glycoprotein IIb/IIa. In addition, they exhibit good dialytic properties and can be cleared using filters. During cardiopulmonary bypass, the amount of NM entering the human body is very small. NM is eliminated from the body very quickly, with a half-life of 8 minutes [5]. NM can effectively fill the gap between heparin and citric acid anticoagulation, providing a new choice for patients who typically undergo heparin-free anticoagulation.

This retrospective study investigated the therapeutic effects of nafamostat in patients undergoing maintenance hemodialysis who experienced conjunctival hemorrhage. Six patients were divided into three groups: heparin-free, nafamostat (diabetic patients), and nafamostat (non-diabetic patients) anticoagulation, and the hemoglobin level, platelet count, blood pressure, pre-dialysis urea nitrogen level, and dialysis adequacy were compared between the groups. The pre- and post-dialysis hemoglobin level, platelet count, and electrolyte levels were compared in patients administered nafamostat, and the adverse reactions, complications, and filter lifespans were compared between the heparin-free and nafamostat groups.

The baseline characteristics were not significantly different between the groups. The hemoglobin level and platelet count did not change significantly after dialysis in patients in the nafamostat group, and no patient experienced high blood potassium or low blood sodium levels. One patient in the heparin-free group experienced filter clotting. Patients in the nafamostat group did not have increased conjunctival bleeding compared to those in the non-heparin group, and no complications including allergic reactions, nausea and vomiting, cardiac arrest, hyperkalemia, hyponatremia, and thrombocytopenia were reported in the nafamostat group (Supplementary material). Patients in the NM groups were satisfied with the choice of anticoagulation therapy (Table 1).

**Table 1:** Outcomes.

		Nafamostat group		
	Heparin-free group	DM	non-DM	*P* value
	P1/P2	P3/P4	P5/P6	
Hemodialysis filters or tubing coagulation	1/0	0/0	0/0	/
Patients with increased conjunctival bleeding volume(numbers)	0/0	0/0	0/0	/
Recovery time of conjunctival hemorrhage (days)	4/5	2/4	4/4	*P *> 0.05

Abbreviations: P1/P2/P3/P4/P5/P6 = Patient1/2/3/4/5/6, DM = diabetes mellitus

The results of our study indicate that NM has advantages as an anticoagulant for patients undergoing maintenance hemodialysis with conjunctival bleeding. Patients in the NM group had no significant changes in hemoglobin level, platelet count, potassium level, or sodium level before and after HD in this study, and no patients in this group required HD filter or tube coagulation. By contrast, one patient in the heparin-free group required replacement of the HD filter and tubing due to coagulation. In our studies, the use of NM did not increase conjunctival bleeding or prolong the recovery time, patient satisfaction was high in the NM group, and no patient experienced complications or adverse reactions.

In conclusion, NM is a new anticoagulation attempt for patients with conjunctival hemorrhage who undergo maintenance HD. However, the number of patients included in this study is small. Therefore, more research with larger patient populations is necessary to further demonstrate the safety and effectiveness of NM in patients undergoing HD.

## Supplementary Material

sfae175_Supplemental_File
