# Supplementary material for: Nafamostat mesylate has advantages as an anticoagulant for patients undergoing maintenance hemodialysis with conjunctival bleeding
Source: Clin Kidney J. 2024 Jun 13;17(7):sfae175. doi: 10.1093/ckj/sfae175 (PMC11252668; doi:10.1093/ckj/sfae175)

Supplementary

Supplementary 1. Patient characteristics

		Heparin free group	Nafamostat group		P value
			DM	non-DM	
Age (years)		61.0±9.8	62.0±32.5	53.0±14.1	0.9012
Male/Female		1/1	1/1	1/1	/
Blood pressure (mmHg)	SBP	136.5±10.6	139.5±4.9	125.0±4.2	0.2512
	DBP	68.5±1.1	64.1±17.2	72.0±19.3	0.8766
Bun		15.3±7.9	27.0±0.6	20.1±0.4	0.1777
spKTV		1.3±0.07	1.26±0.01	1.35±0.09	0.4646
Hb (g/l)		101.0±6.3	95.0±4.2	98.0±22.6	0.8982
Plt (×1000/L)		212.2±84.3	165.5±86.9	181.0±55.1	0.8351

Abbreviations: DM = diabetes mellitus, SBP = systolic blood pressure, DBP = diastolic blood pressure, BUN = blood urea nitrogen, Hb = hemoglobin, PLT= platelet.

Supplementary 2. Coagulation function changes before and after NM administration

No.	Project	Result1	Result2	Unit	Reference	Standard code display
1	Prothrombin time measurement	12.5	13.8 ↑	sec	10.5 ~ 13.7	PT
2	Prothrombin activity	88.7	76.8	%	70.0 ~ 150.0	PTA
3	International standardized ratios	1.12	1.24 ↑		0.80 ~ 1.20	INR
4	Activated partial thrombin time	27.3	58.7 ↑	sec	25.0 ~ 31.3	APTT
5	Fibrinogen concentration	6.17	/	g/L	1.50 ~ 3.50	FIB
6	Thrombin time	14.6	/	sec	14.0 ~ 21.0	TT
7	D dimer	1.08	/	mg/L	0.00 ~ 0.55	D-Dimer
8	Fibrin degradation products	2.8	/	mg/L	0.0 ~ 5.0	FDP

Abbreviations: Result1=Coagulation function before hemodialysis; Result2=Coagulation function after 2 hours of use of nafamostat

Supplementary 3. Changes in blood parameters in the NM groups

	Nafamostat group		P value
	Before using Nafamostat	After using Nafamostat	
Hb (g/l)	96.5±13.4	94.5±7.3	0.8020
Plt (×1000/L)	173.2±60.1	159.0±59.9	0.7485
Blood potassium(mmol/l)	4.7±0.68	4.3±0.38	0.3910
Blood sodium(mmol/l)	141.5±1.54	139.0±2.25	0.1164

Abbreviations: DM = diabetes mellitus, Hb = hemoglobin, PLT= platelet.

Supplementary 4. Complications and adverse reactions

Patients with complications(numbers)	Heparin free group	Nafamostat group		Total
		DM	non-DM	
Allergic reactions	0(2)	0(2)	0(2)	(0)6
Nausea or vomiting	0(2)	0(2)	0(2)	(0)6
Cardiac arrest	0(2)	0(2)	0(2)	(0)6
Hyperkalemia	0(2)	0(2)	0(2)	(0)6
Hyponatremia	0(2)	0(2)	0(2)	(0)6
Thrombocytopenia	0(2)	0(2)	0(2)	(0)6
Hemodialysis filters or tubing coagulation	1(2)	0(2)	0(2)	(1)6

Abbreviations: DM = diabetes mellitus

Supplementary 5. Figure captions

Figure1. Patients undergoing maintenance hemodialysis require anticoagulants that do not contain heparin. (A-B) Data of male patients in the heparin-free anticoagulation group are shown. Approximately five days after conjunctival bleeding, the bleeding gradually improved. (C-D) Data of a female patient in the heparin-free anticoagulation group are shown. Approximately four days after conjunctival bleeding, the bleeding gradually improved.

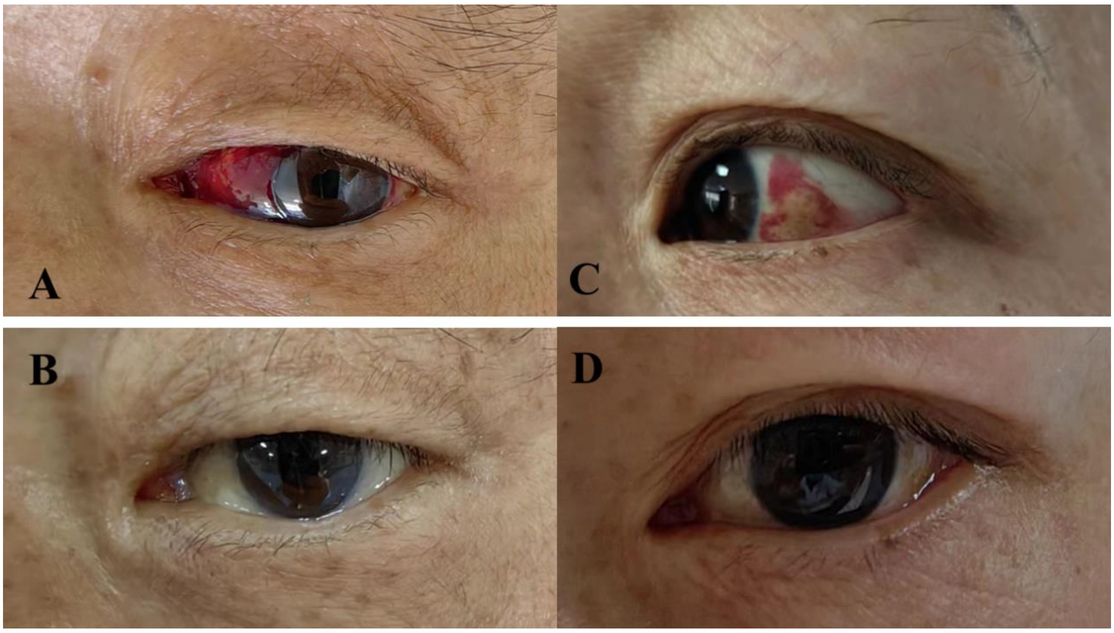

Figure2. Nafamostat was administered to patients with diabetes undergoing maintenance hemodialysis. (A-B) Data of male patients in the nafamostat anticoagulation group are shown. After approximately three days of conjunctival bleeding, the bleeding gradually improved. (C-D) Data of a female patient in the nafamostat anticoagulation group are shown. Approximately four days after conjunctival bleeding, the bleeding gradually improved.

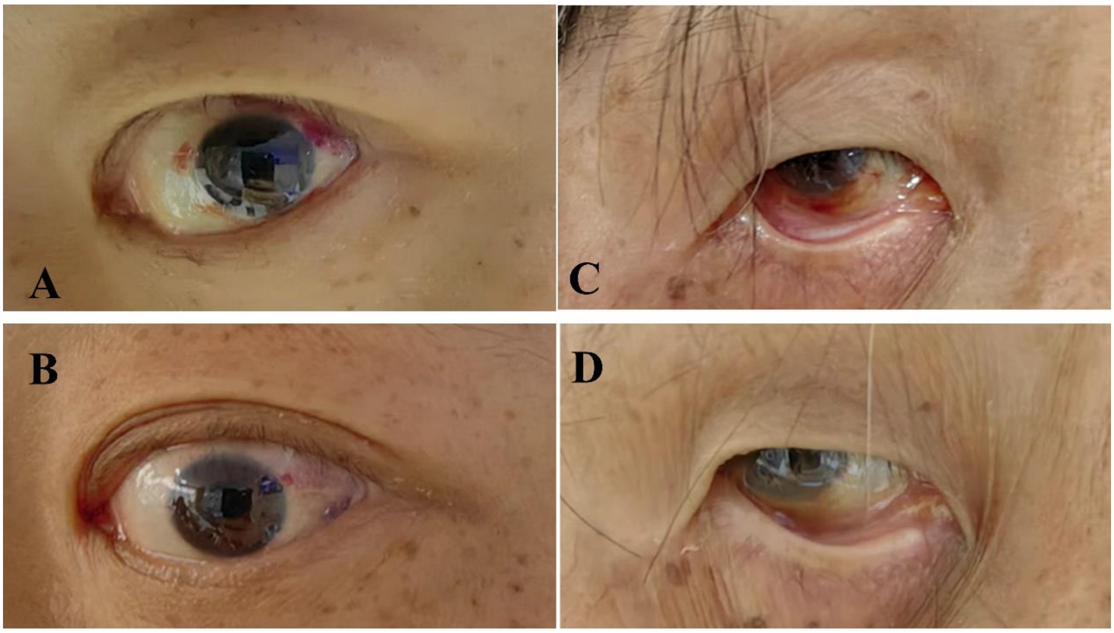

Figure 3. Nafamostat was administered to patients without diabetes undergoing maintenance hemodialysis. (A-B) Data of male patients in the nafamostat anticoagulation group are shown. After approximately three days of conjunctival bleeding, the bleeding gradually improved. (C-D) Data of a female patient in the nafamostat anticoagulation group are shown. Approximately four days after conjunctival bleeding, the bleeding gradually improved.

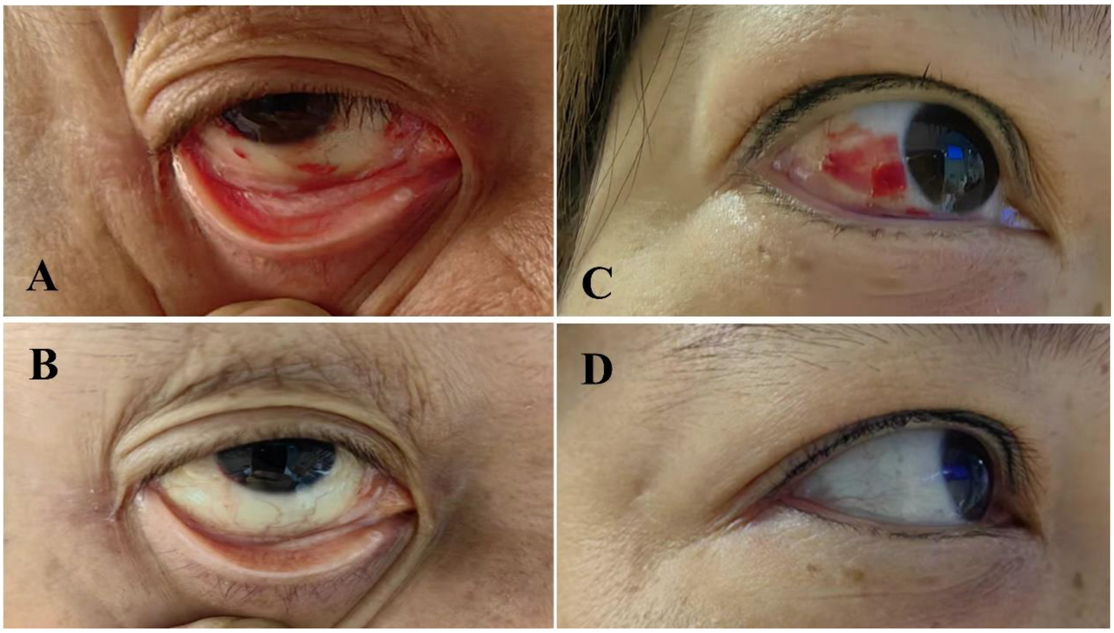

Supplement: sfae175_Supplemental_File [file sfae175_supplemental_file.pdf]
